# Public perception of coastal habitat loss and habitat creation using artificial floating islands in the UK

**DOI:** 10.1371/journal.pone.0224424

**Published:** 2019-10-31

**Authors:** Jessica Ware, Ruth Callaway

**Affiliations:** Biosciences, Swansea University, Swansea, United Kingdom; Macquarie University, AUSTRALIA

## Abstract

Eco-engineering and the installation of green infrastructure such as artificial floating islands (AFIs), are novel techniques used to support biodiversity. The European Convention on Biological Diversity highlighted the development of green infrastructure as a key method of enhancement in degraded habitats. Research specifically on AFIs in marine environments has largely focused on their ecological functioning role and engineering outcomes, with little consideration for the social benefits or concerns. The aim of this study was to gain an understanding of public perception of coastal habitat loss in the UK and AFIs as a method of habitat creation in coastal environments. This was achieved via a survey, consisting of six closed and two open questions. Of the 200 respondents, 94.5% were concerned about the loss of coastal habitats in the UK, but less than a third were aware of habitat restoration or creation projects in their area of residence. There was a positive correlation between proximity of residency to the coast and knowledge of habitat restoration or creation projects. The majority of the respondents understood the ecological functioning role of AFIs and 62% would preferably want successful plant growth and avian species utilising the AFI. Nearly a third of the respondents had concerns about AFI installations, such as the degradation of the plastic matrix, long term maintenance and disturbance of native species. Despite 90.9% of the respondents supporting the installation of AFIs, the concerns of the public must be addressed during the planning stages of any habitat creation project.

## Introduction

By 2025, more than 75% of the human population is estimated to live within 100km of the coast [1–6]. Currently, 14 of the World’s largest cities occupy coastal regions [4], associated with extensive infrastructure to support commercial, residential and recreational developments [1,7–12]. Due to the risk of flooding and erosion caused by rising sea levels and severe storms, densely populated areas require protection via coastal defences such as sea walls, groynes and revetments [1,7,13–17]. The combined impact of coastal ‘armouring’ and marine urban sprawl has caused increasing spatial disconnection of coastal habitats, habitat degradation and alterations to natural community assemblages [1,18–22]. Coastal wetlands for example, are considered one of the most threatened ecosystems, with up to 50% of global saltmarsh recorded as either lost or degraded [23–26]. Avian species are reliant on coastal habitats for nesting, foraging and roosting and are increasingly under threat, due to rising sea levels and proposed coastal infrastructure [27]. Fish larvae dispersal and recruitment can also be disrupted by coastal infrastructure, which causes fluctuations in current patterns and sediment loading [28,29]. The European Convention on Biological Diversity aims to prevent any further loss of biodiversity and ecosystem services in Europe by 2020, with the support of novel techniques such as eco-engineering and green infrastructure [30–32]. The United Kingdom (UK) Post–2010 Biodiversity Framework intends to meet these international obligations, utilising biodiversity enhancement methods where appropriate [33].

Eco-engineering refers to the modification of planned or existing structures to become multifunctional [8,34–36]. The process integrates ecological theory with the design of a proposed structure, either during the construction or post construction phase [37]. For example texture can be added to a sea wall via small indents, larger pits or water holding features, such as flower pots [32,38–40]. In highly modified marine ecosystems such as marinas and docks, eco-engineering offers a means of enhancing existing or planned structures to benefit local biodiversity, while maintaining the integral anthropogenic function of the structure [41–43].

**Fig 1:**
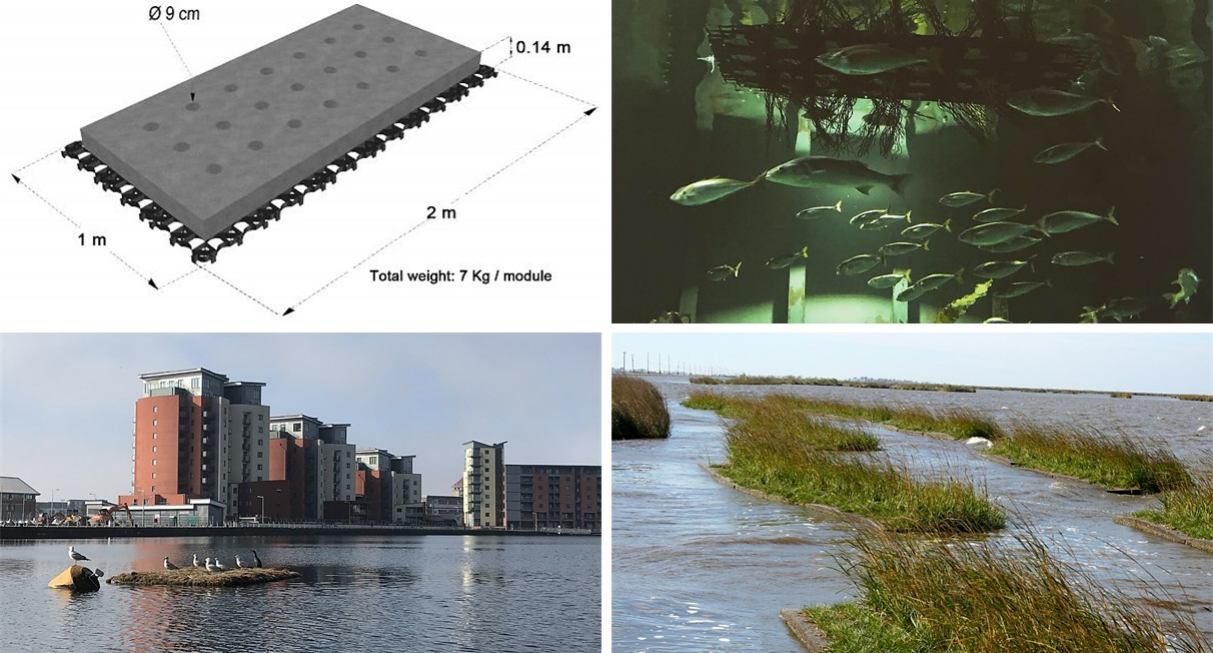
Artificial floating island (AFI) unit and existing installations and research. *Top left*–Schematic diagram of a 2m^2^ matrix unit, commercially sold as ‘biohavens’. These AFIs consist of a non-woven plastic matrix, integrated connection grid and polyurethane foam [53]; *top right*–AFI installed in a controlled experiment at Bristol Aquarium, with 13 native, marine vertebrates; *bottom left*–AFI installed in a saline dock in Swansea known as Prince of Wales Dock; and *bottom right*–Linear arrangement of AFIs used on the coast of Louisiana, USA, for wave absorption and to reduce coastal erosion [60].

AFIs, also referred to as floating treatment wetlands, biohavens and floating ecosystem modules, offer an alternative eco-engineering method [44,45]. These small-scale floating structures should not be confused with the larger land reclamation activities occurring around the world and proposals for floating cities to support population growth and climate migration [46,47]. In the UK, they are commercially sold by companies that provide eco-engineering solutions for silt management, plastic pollution, wastewater treatment and habitat creation. They broadly consist of a buoyant mat, planting media and emergent vegetation [48–51]. The design referred to in this study (Fig 1, *top left*), consists of a non-woven recycled plastic matrix, an integrated connection grid providing structure and closed cell polyurethane foam for buoyancy [52,53]. With established plants grown on coir matting, AFIs support a localized ecological community within the submerged roots and on the surface of the structure itself; these include algal communities, macroinvertebrates and epibiotic species [49,54]. They have largely been installed in deteriorated and over-modified freshwater habitats to improve water quality, via the removal of suspended solids and organic matter, and biosynthesis of nutrients, effectively purifying the surrounding water body [48,49,55–58]. However, interest in the use of AFIs in coastal environments has increased and is the key focus of this study [59].

Over 300 AFIs have been utilised by the Royal Society for the Protection of Birds (RSPB) to provide breeding grounds and roosting sites for divers, gulls, terns, waders and wildfowl species, within coastal wetlands in the UK [61]. Their use extends to conservation projects in San Leandro Bay Oakland, California, to provide tidal refuge habitat for the California Ridgeway’s rail (*Rallus obsoletus obsoletus*) during inundation periods of the natural wetland habitat [57]. Floating structures also promote the formation of biofouling communities [44,62,63], increasing productivity and nutrient availability via deposition of organic matter within the local environment. This can attract higher trophic species such as fish, elevating the local species diversity [63–65]. For example juvenile common two-banded sea bream (*Diplodus vulgaris*) have been associated with artificial structures in high abundances, utilising installed ‘biohuts’ that add complexity to the localised habitat [29]. In Swansea, three AFIs have been installed in inshore marine habitats to assess the successful establishment of vegetation and their utilisation by birds, fish and invertebrates (Fig 1, *bottom left*). However, there currently is a lack of understanding of the public perception of AFIs, which could impact on the success of future installation projects [35,66–68].

Public awareness and perception of both national and international scale environmental concerns is important, as it influences acceptance of environmental policies and positive behavioural change within society [69,70]. Understanding the relationship the public currently have with marine ecosystems will enable the identification of any misconceptions of environmental issues and highlight the issues of concern [71]. With a better understanding of successful and failed processes of scientific communication, future environmental management and policy strategies can be improved, encouraging public support. Incorporating public awareness and citizen science campaigns into environmental conservation can positively contribute to the success of achieving new, conservation objectives [72–74]. Previously, the importance of stakeholder engagement has been highlighted during the installation of artificial reefs off the west coast of Scotland and southern Portugal [75,76]. In a number of studies worldwide, the majority of the respondents supported eco-engineering initiatives that enhanced the conservation of biodiversity [35,67,68]. However, awareness and knowledge of eco-engineering initiatives tends to be lower in Europe compared to America and Australia [67].

In the UK, public perception research has focused on the general marine environment and its protection from global concerns such as climate change [72,77–79], managed realignment [80,81], beach aesthetic and selection [82] and offshore wind farms [83]. It is important that similar information is gained on the public perception of eco-engineering methods, such as AFIs.

This study aimed to gain an understanding of the perceived importance of coastal habitat loss in the UK, in comparison to other environmental issues. Further, the study aimed to obtain information on the public’s understanding of AFIs and any concerns related to AFI installations. The objectives of the survey were to assess whether the public were: (1) concerned about the loss of coastal habitats in the UK; (2) aware of local habitat restoration or creation projects; (3) aware of the ecological functioning role of AFIs; and (4) supportive of AFI initiatives as a method of habitat creation within coastal environments. Further, the study aimed to assess whether public awareness correlated with proximity of residency from the coast. The results of this study will help inform stakeholders planning on installing AFIs in UK coastal environments on public opinion and best practice before and during the AFI installation.

## Methods

### Survey design

The survey consisted of eight questions, subdivided into two themes: coastal habitats and AFIs (Table 1). The survey included questions with 5-point Likert scale answers, binary and multiple choice. It was restricted to six closed questions and two open questions, with an average completion time of 3 minutes, thus maximising participation. No background information was provided prior to the respondent completing the survey. Question 1 was limited to five factors for simplicity and the factors selected were all environmental concerns prevalent in the UK. In terms of personal information, only distance that the respondent lived from the coast was determined. Other demographic information was not collected in this survey, such as age and occupation, as these details were not required to meet the study objectives. However, more detail about the location of residency was inferred from Question 3, addressing awareness of habitat restoration initiatives and assuming that participants had greater knowledge of projects in their local area. Question 5 addressed a common issue associated with high numbers of wildfowl and maintaining plant growth on AFIs. Additionally, AFIs can be specifically installed without vegetation to attract certain avian species that require only substrate for breeding [61,84].

**Table 1:** The complete survey consisting of 8 questions.

**Section 1: Coastal habitats**		
**Questions**	**Possible answers**	
1. Which of the following factors do you think are negatively impacting on the health of coasts in the UK? Rank each factor by importance. Urbanisation/ Coastal Developments, Flooding, Invasive species, Plastic pollution and Habitat loss.	Very important, Fairly important, Important, Slightly important or Not at all important.	
2. Are you concerned about the loss of coastal habitats in the UK, such as beaches, coastal wetlands and saltmarsh?	Yes, No or Not sure.	
3. Are you aware of any habitat restoration or creation projects in your area like artificial floating islands or wildflower planting? If yes, any further details of the type of project and in what location can be added here.	Yes or No.	
**Section 2: Artificial floating islands**		
**Questions**		**Possible answers**
4. Artificial floating islands consist of a recycled plastic matrix and growing medium, that plants are able to grow roots through. They are often installed in lakes and rivers. What do you think artificial floating islands are installed for? Tick any answers that you think are correct.	Aesthetic, To create habitat and support biodiversity, To support boating activity, To improve water quality, To collect litter or Other.	
5. On some occasions it is difficult to maintain both plant growth and bird use. Which of the following scenarios would you prefer if an island were installed in your local area?	Bird activity and no plants, Plants and fencing with roots growing through the island for fish, Plant growth but not fully covering the island and bird activity or Not sure.	
6. Would you have any concerns about the installation of an artificial floating island?	Open question.	
7. Would you support future installations of artificial floating islands or other habitat creation projects along the coast?	Yes, No or Not sure.	
8. How far from the coast to do live?	1 mile, 5 miles, 10 miles or 20 miles +.	

### Survey collections

The target demographic was members of the public living in the UK, aged 18 or above. One respondent living in the Netherlands completed the survey and was included in the analysis. The survey was self-administrated using the survey tool ‘Survey Monkey’ (https://www.surveymonkey.com) and went live on 27^th^ January 2019. The survey was live for 68 days, until 5^th^ April 2019. The survey was circulated on social media platforms such as Facebook and Twitter and members of the public were approached in Bristol Aquarium and Swansea. The survey was also circulated via community forums such as such as ‘Maritime Quarter Residents Association’ and ‘Uplands and Brynmill community forum’, to gain information on the opinion of local residents, who may have observed the AFIs in Swansea. A total of 200 surveys were collected during the 68 days that the survey was live (online, n = 170; in person, n = 30). The information provided during the online surveys and in person was the same, minimising any bias results. Swansea University ethics committee approved research conducted in this study (SU-Ethics-Student-030719/1106).

### Data analysis

Descriptive statistics were used to summarise results from each question of the survey. Chi squared tests were used to assess whether there was a relationship between the distance the respondent lived from the coast and their (1) concern of coastal habitat loss; (2) awareness of habitat restoration and creation projects; (3) awareness of AFIs and their ecological functioning role; and (4) concerns related to AFIs being installed. Comments that addressed concerns about AFI installations (Question 6; Table 1) were organised into categories appropriately. Statistical tests were completed using R 3.6.0 statistics software.

## Results

Of the 200 respondents, 29.5% (n = 59) lived within 1 mile of the coast, 23% (n = 46) within 5 miles, 17.5% (n = 35) within 10 miles and 30% (n = 60) greater than 20 miles.

### Coastal habitats

**Fig 2:**
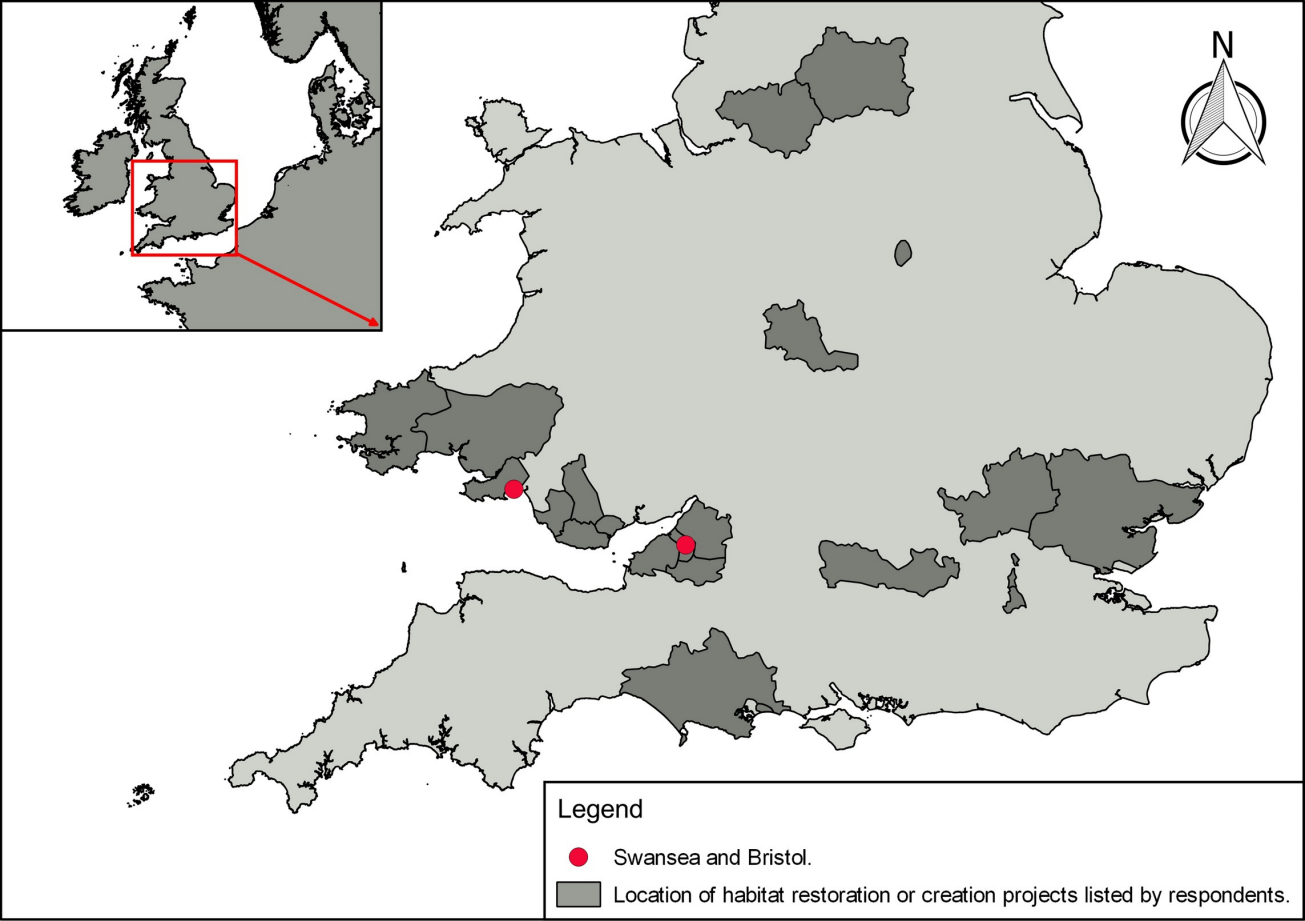
The perceived importance of factors negatively impacting on the health of UK coasts.

The majority of respondents considered plastic pollution (77.8%, n = 154) and habitat loss (70.9%, n = 139) to be very important factors affecting the health of coasts in the UK (Fig 2). Urbanisation was also considered to be a very important factor by 43.2% of the respondents (n = 86). There was no significant relationship between perceived importance of coastal habitat loss and proximity of residence to the coast (χ^2^ = 2.86, d.f. = 3, p = 0.41, n = 200). Less than a third of the respondents considered flooding (28.4%, n = 55) and invasive species (24.2%, n = 47) to be very important factors affecting the health of coasts in the UK. Three of the factors were perceived as not important at all. These were invasive species (5%, n = 9), flooding (4%, n = 7) and urbanisation/coastal developments (1%, n = 2).

**Fig 3:**
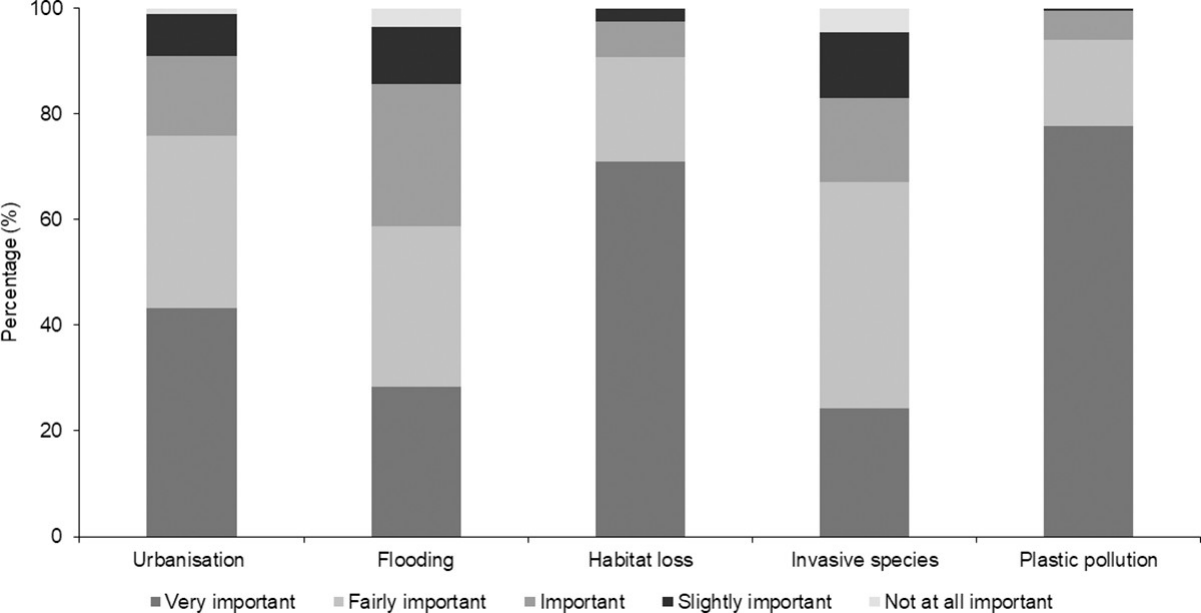
The location of habitat restoration or creation projects listed by the respondents of the survey (n = 34). The projects mentioned by respondents were located in 23 counties in England and Wales. Each project is represented by county it is located in [85,86].

The majority of respondents were concerned about the loss of coastal habitats in the UK (94.5% n = 189). Under a third of the respondents (28.5%, n = 57) were aware of habitat restoration or creation projects in their area of residence and this was dominated by respondents living within 1–5 miles of the coast (70%). There was a significant relationship between the respondents’ awareness of habitat restoration and creation projects and the proximity of residence from the coast (χ^2^ = 8.95, d.f. = 3, p = 0.02, n = 200). The respondents that provided further detail to Question 3 (n = 34) mentioned projects located in South Wales and England (Fig 3) and 52% of the schemes were related to marine environments, rather than terrestrial or freshwater habitats.

### Artificial floating islands

As the respondents could give multiple answers on the perceived purpose of installing an AFI (Table 1, Question 4), there were 385 responses; 306 understood the ecological functioning role of AFIs (‘to create habitat and support biodiversity’ n = 196; ‘to improve water quality’ n = 110). There was no significant relationship in public awareness of the ecological functioning role of AFIs between the four proximity categories (χ^2^ = 3.64, d.f. = 3, p = 0.30, n = 200).

**Fig 4:**
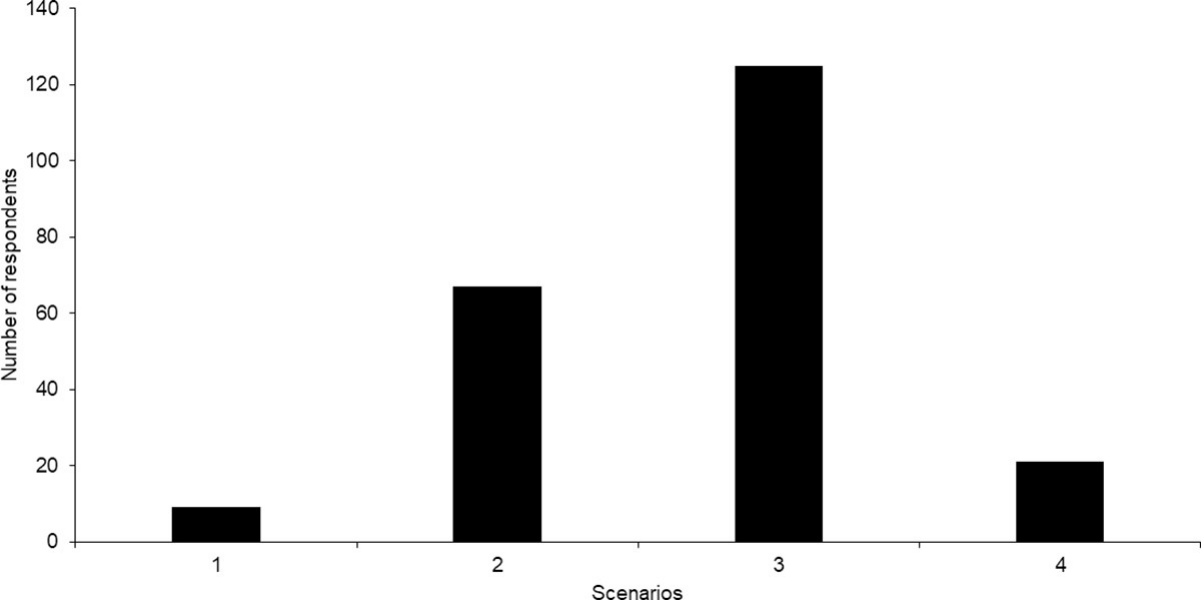
The respondents’ preference of an installed artificial floating island in their local area based on five scenarios. (1) Bird activity and no plants; (2) Plants and fencing, with roots growing through the island for fish; (3) Plant growth, but not fully covering the island and bird activity; and (4) Not sure.

The majority of the public surveyed preferred to have both successful plant growth and birds utilising an AFI (62%, n = 125, Fig 4). One third of the respondents preferred the installation of an island with successful plant growth, maintained by the inclusion of fencing (33%, n = 67). High levels of bird activity with no plants growing was the least popular response (4%, n = 9).

**Fig 5:**
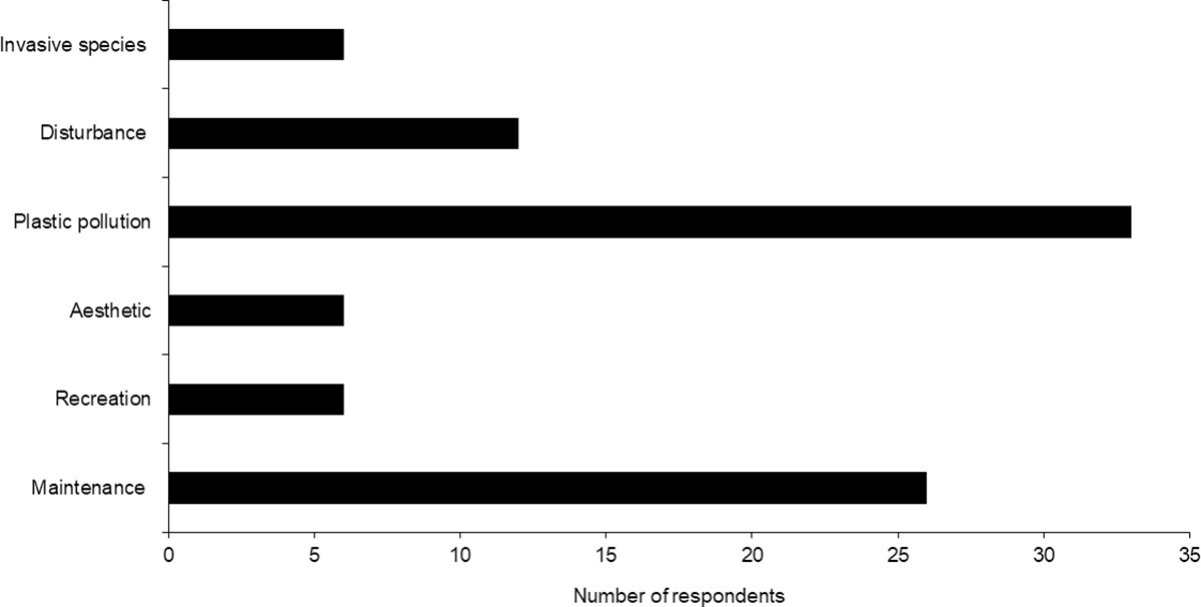
The number of concerns raised by respondents. These have been categorically organised into maintenance, recreation, aesthetic, plastic pollution, disturbance and invasive species.

Question 6 of the survey allowed the respondents to voice any concerns regarding AFI installations on the coast; 33% of the 200 (n = 66) chose to comment on their concerns. These were broadly categorised into maintenance, recreation, aesthetic, plastic pollution, disturbance and invasive species concerns (Fig 5). The definition of each term based on the respondents’ answers are outlined in Table 2.

**Table 2:** Definition of the six concerns listed by respondents in Question 6 of the survey.

Concern	Definition
Maintenance	Damage or detachment of the island during severe weather or as a result of vandalism.
Recreation	Disrupt boating, kayaking or surfing activity on the coast.
Aesthetic	It is unnatural and a potential eyesore.
Plastic pollution	Degradation of the plastic matrix into the water body.
Disturbance	Noise pollution during installation and impact on natural processes.
Invasive species	Encourage the presence or spread of a non-native species that could cause damage to the ecosystem.

Plastic pollution (n = 33) and the long-term maintenance (n = 26) of an installed AFI were the key areas of concern by the respondents of the survey (Fig 5). The majority of the respondents would support the future installation of AFIs along the coast (90.9%, n = 181), with the remaining respondents either unsure or against the method of habitat creation.

## Discussion

Artificial structures are proliferating in marine environments in the form of coastal defences [13,14,17] and infrastructure to support shipping, transport, commercial, recreational and residential developments [1,7–12]. Current legislation including the European Convention on Biological Diversity and the UK Post-2010 Biodiversity Framework, address that novel techniques such as eco-engineering have a role to play to prevent any further loss of biodiversity and ecosystem services caused by anthropogenic activities [30–32,87]. Alongside meeting legislative targets, it is also important to engage with the public on environmental issues and conservation approaches that could be introduced. Without public engagement, the awareness and public support of future projects cannot be guaranteed. This study aimed to gain an understanding of the public’s perception of coastal habitat loss and AFIs as a habitat creation method.

The majority of participants of this survey were concerned about the loss of coastal habitats in the UK and consider plastic pollution, habitat loss and urbanisation as very important factors negatively impacting on the coast (Fig 2). Due to the release of documentaries such as ‘A Plastic Ocean’ in 2016 and ‘Blue Planet II’ in 2017, public awareness has increased substantially on the impacts of litter and specifically, non-biodegradable material in ocean ecosystems. The UK public also demonstrated an understanding of the deterioration of marine environments in a previous study, where 95.8% of respondents considered marine habitats to be of ‘fair to poor’ health [72,78]. Pollution and climate change are consistently mentioned as the most concerning environmental issues for members of the public, in the UK and abroad [35,77,88]. In this survey, coastal urbanisation, flooding and invasive species were perceived as less important factors by some respondents (Fig 2). This could be due to a lack of understanding of secondary impacts of developments, such as light and noise pollution and fluctuating hydrodynamics that can result in flooding. The importance of flooding to the respondent can also be governed by personal experience [89]. The individuals’ socio-economic status linked to education and occupation and their specific motivations and interests, have also been identified as factors that drive awareness of environmental issues [77,90]. These details were not included as part of this survey, as the information was not required to meet the study research objectives. However, this does limit comparisons to other public surveys.

The majority of the public desire greater protection and conservation of the UK marine environment, from fishing and other damaging, exploitative practices [72]. However, as part of this survey under a third of the respondents were aware of habitat restoration or creation projects in their area. The respondents that did mention restoration and/or creation projects mostly lived within 1–5 miles of the coast and 52% of the schemes were related to marine environments, rather than terrestrial or freshwater habitats. Examples of schemes mentioned across all habitat types included: dune slack management in Kenfig National Nature Reserve, Bridgend, to promote early succession of orchids; creating habitats for common kingfisher (*Alcedo atthis*) populations in the Lee Valley, Essex, via river management and; habitat restoration at Saltwells Local Nature Reserve, Dudley (Fig 3). The focus on marine conservation and policy could be a direct result of greater national awareness, personal interest based on residential location or occupation. The correlation between proximity to the coast, marine conservation and policy knowledge was discovered during a large scale survey in the United States [77,90]. However, this outcome could also be a result of the marine focus of the survey. To reduce potential bias towards marine projects, wildflower planting was also mentioned as a terrestrial habitat restoration and/or creation method in Question 3. The respondent was also asked to mention projects within their local area (Table 1).

For future research, more detailed demographic information would be desirable to gain deeper insight into relationships between social and economic background with views on marine conservation awareness and AFIs.

In this survey, the majority of respondents showed an understanding of the ecological functioning role of AFIs. This could be linked to a positive shift in perception in the UK of the importance of wetland biodiversity and support towards wetland restoration [91]. Overall, the survey confirmed that the public preferred a vegetated island utilised by birds (Fig 4). Within urban environments green landscapes play a significant role in health and mediating the stresses of daily life [92,93]. This could have contributed to the respondents’ positive association with vegetation growth on the AFIs. Water quality of natural wetlands, the presence of emergent vegetation and trees and habitat value to local wildlife, were factors viewed as important in assessing wetland health in Australia [94]. There is however, evidence that a lack of understanding of ecological values is linked to a negative view of wetlands [94–96].

Public and stakeholder perception studies of artificially created habitats have largely focused on benthic habitats including artificial reefs, concrete flowerpots used in the intertidal zone and coastal defence structures [35,67,68,75,76,97]; therefore, limiting comparisons of the results from this study. In a preliminary study, stakeholders including engineering and ecological consultants, academics and statutory bodies unanimously supported the implementation of multi-functional artificial structures, which prioritised ecological benefits within coastal environments [66]. The study also highlighted that ‘education and outreach’ was one of the lowest assigned considerations by stakeholders, while a greater evidence base of the ecological benefits was seen as desirable. This illustrated the importance of accessible research and a strong evidence base for stakeholders [12]. It also demonstrated the lack of importance placed on public engagement by stakeholders, which could be limiting future public support of the implementation of eco-engineering and artificial habitat creation projects.

Nearly a third of the respondents had concerns about the installation of AFIs in the marine environment. These concerns largely focused on the future degradation of the AFI matrix and potential for the islands to become plastic pollution (Fig 5). Additionally, the public were concerned about the long-term maintenance and aesthetic of the island; ‘would it look unnatural and therefore un-aesthetic?’, ‘how will they be maintained?’, and ‘would the plastic in the matrix enter the food chain?’. Other comments were related to the potential disturbance of commercial and recreational boating, surfers and native wildlife. During the planning stages of an AFI installation, it is important that research and monitoring is undertaken by the individual or company responsible, on the environmental conditions of a proposed island location. This includes factors such as average wind speed, water velocity and tidal height (if applicable). In addition, salinity and pH should be assessed as certain metals are susceptible to corrosion, based on the surrounding water chemistry. This information will aid decisions on the appropriate size, configuration and method of installation of an AFI, that minimises disruption of native fauna and ensures it is securely installed. Research and open communication with potential stakeholders and members of the public, will also ensure that no recreational activities are disrupted by the installed AFI.

AFIs have an approximate life span of 20 years and this varies depending on its location [50]. As most AFIs are installed in ponds, reservoirs and rivers, case studies of islands exposed to waves, tides, marine biofouling and saline conditions are limited. Laboratory experiments have demonstrated that the size and configuration of the AFI determines the force (kilonewton, kn) exerted on the islands structure. Prior to the installation of an AFI, a maintenance and potential disposal plan should be established and made publicly accessible. This will ensure the long-term success of an AFI and reassure local residents that the island will be maintained and disposed of appropriately, to prevent potential degradation of the plastic matrix. If the AFI is installed where invasive species are present, the island should not be translocated to prevent the potential spread of invasive species.

In conclusion, the majority of the respondents would support the installation of AFIs along the coast, as they recognised coastal habitat loss as an important environmental issue. The successful establishment of plants and positive benefits to local wildlife, were equally important factors valued by respondents. There were concerns regarding the longevity of an artificially created habitat, which must be rectified with thorough strategic planning and appropriate aims, based on the location of the proposed AFI installation. Further research is required on socio-economic factors that could be influencing public awareness of habitat loss and artificially created habitats within urban ecosystems.

## Supporting information

S1 Appendix Dataset of the survey. Includes the 200 respondents’ answers to the eight questions of the survey that was open from 27^th^ January– 5^th^ April 2019.
(XLSX)

S2 Appendix Survey questions. The eight survey questions answered by respondents.
(PDF)
