# Supplementary material for: Public perception of coastal habitat loss and habitat creation using artificial floating islands in the UK
Source: PLoS One. 2019 Oct 31;14(10):e0224424. doi: 10.1371/journal.pone.0224424 (PMC6822727; doi:10.1371/journal.pone.0224424)
Supplement: S2 Appendix — The eight survey questions answered by respondents. (PDF) [file pone.0224424.s002.pdf]

Coastal Habitats and Artificial Floating Islands

Section 1: Coastal Habitats

1) Which of the following factors do you think are negatively impacting on the health of coasts in the UK? Rank each factor by importance. (1 = Very important, 2 = Fairly important, 3 = Important, 4 = Slightly important, 5 = Not at all important)

- ☐ Urbanisation/ Coastal Developments
- ☐ Flooding
- ☐ Invasive species
- ☐ Plastic pollution
- ☐ Habitat loss

2) Are you concerned about the loss of coastal habitats in the UK, such as beaches, coastal wetlands and saltmarsh?

- ☐ Yes
- ☐ No
- ☐ Not sure

3) Are you aware of any habitat restoration or creation projects in your area like artificial floating islands or wildflower planting?

- ☐ Yes
- ☐ No

If yes, any further details of the type of project and in what location can be added here.

Section 2: Artificial Floating Islands

Artificial floating islands consist of a recycled plastic matrix and growing medium, that plants are able to grow roots through. They are often installed in lakes and rivers.

4) What do you think artificial floating islands are installed for? Tick any answers that you think are correct.

- ☐ Aesthetic
- ☐ To create habitat and support biodiversity
- ☐ To support boating activity
- ☐ To improve water quality
- ☐ To collect litter

Other …………………………………..

5) On some occasions it is difficult to maintain both plant growth and bird use. Which of the following scenarios would you prefer if an island were installed in your local area?

- ☐ Bird activity and no plants
- ☐ Plants and fencing with roots growing through the island for fish
- ☐ Plant growth but not fully covering the island and bird activity.
- ☐ Not sure

6) Would you have any concerns about the installation of an artificial floating island?

7) Would you support future installations of artificial floating islands or other habitat creation projects along the coast?

- ☐ Yes
- ☐ No
- ☐ Not sure

8) How far from the coast to do live?

- ☐ 1 mile
- ☐ 5 miles
- ☐ 10 miles
- ☐ 20 miles +
